# Subjective health complaints in adolescent victims of cyber harassment: moderation through support from parents/friends - a Swedish population-based study

**DOI:** 10.1186/s12889-015-2239-7

**Published:** 2015-09-23

**Authors:** Maria Fridh, Martin Lindström, Maria Rosvall

**Affiliations:** Department of Clinical Sciences, Social Medicine and Health Policy, CRC, Jan Waldenströmsgata 35, Malmö University Hospital, Lund University, SE-205 02 Malmö, Sweden; Centre for Economic Demography, Lund University School of Economics and Management, SE-220 07 Lund, Sweden

**Keywords:** Cyber harassment, Subjective health complaints, Adolescent, Sweden, Parental support, Friend support, Population study

## Abstract

**Background:**

Victimization in cyberspace has emerged as a new public health issue among the young. The main purpose of this study was to analyze associations between cyber victimization defined as cyber harassment (CH) (a somewhat broader concept than cyberbullying) and subjective health complaints (SHC), to study whether these associations were modified by parental/friend support (measured as communication), and to explore the influence of traditional bullying victimization (TBV) on the association between CH and SHC.

**Methods:**

The study population consisted of 8544 students in 9th grade (around 15 years old) who participated in the 2012 Scania public health survey of children and adolescents. The survey was a cross-sectional total-population study conducted in school, with a response rate of 83 %.

Main and interaction (stress-buffering) effects of social support on the relationship between CH and SCH were investigated by hierarchical multiple linear regression analyses, adjusted for potential confounders, including TBV.

**Results:**

The past-year prevalence of CH (once or several times) was 14 % among boys and 20 % among girls. Having been cyber harassed once or several times during the past year was associated with higher levels of SHC, controlling for age, parental occupation, parental origin, daily smoking, intense alcohol consumption, and disability. Among both boys and girls, the associations were stronger for CH occurring several times than for CH occurring only once. Main effects of parental/friend support were seen for both boys and girls, while stress-buffering effects were indicated for boys only. Additional analysis further adjusting for TBV did not change the associations substantially, indicating that CH has an effect of its own on SHC.

**Conclusion:**

Intervention programs aimed at improving the quality of peer and family relationships among children and adolescents might reduce the incidence of both cyber harassment and traditional bullying and lower the prevalence of psychosomatic complaints.

**Electronic supplementary material:**

The online version of this article (doi:10.1186/s12889-015-2239-7) contains supplementary material, which is available to authorized users.

## Background

### Introduction

Cyber victimization has emerged as a new public mental health issue affecting youth today, as expanding use of the Internet and cell phones has provided a new arena for both social interaction and opportunities for abuse [1–4]. In Sweden practically all adolescents have their own cell phones (most often smart phones) and have access to the Internet, where they spend an increasing amount of time [5]. Girls use more social networking sites, chats, and blogs, and more sites where you can upload pictures for public display (e.g., Instagram), while boys play more games and watch more video clips [5].

Cyber victimization can be broadly defined as bullying or harassment performed via electronic means, such as using cell phones or the Internet [4]. However, research has yet to reach consensus on a more precise definition. Extending the concept of traditional bullying into the cyberworld would seem logical [6], but is somewhat problematic [7], as the criteria of traditional bullying—intent to harm, repetition over time, and an imbalance of power between the perpetrator and the victim [6]—are relatively clear-cut in traditional bullying, while the aspects of repetition and power imbalance are more difficult to define in a cyber context [7, 8]. A single online act, such as posting a malevolent picture, may be seen, commented on, and forwarded by many others, which constitutes a repetition, but not necessarily one that involves the original perpetrator [7]. The anonymity of the perpetrator can be viewed as a form of power imbalance, as can the size of the potential audience, the longevity of the message, and the difficulty of escaping from it—there is no safe haven, even at home [1, 3, 7, 8]. It has been argued that the very nature of the Internet implies that all three elements of traditional bullying may be present in a single online interaction [9]. However, it has also been proposed that victimization in cyberspace is less harmful than victimization by traditional bullying as the victim cannot be hurt physically [8].

Estimates of cyber victimization vary widely due to different definitions as well as differences in age group, sampling, methodology, and time frame [1, 10]. Studies with narrow definitions and shorter time frames (past few months) have reported prevalence as low as around 2 % [11, 12], while studies with wider definitions and longer time frames (past year) have reported cyber victimization of more than every fourth adolescent [13]. The Swedish Media Council reported a prevalence of 6 % among boys and 20 % among girls 13–16 years, using the definition of cyber victimization as “Someone having been mean to or bullied you using the Internet or a cell phone during the past year” [14]. Cyber victimization (defined as having been treated in a nasty or hurtful way online during the past 12 months) increased among European children 9–16 years old from 7 % (boys 6 %; girls 8 %) to 12 % (boys 8 %; girls 15 %) between 2010 and 2014 [15]. Traditional bullying victimization (TBV) on the other hand consistently decreased in most countries including Sweden between 1993/94 and 2005/06 [16]. The prevalence of TBV is low in Sweden by international comparison [16–18] however, the associations between TBV and subjective health complaints (SHC) are stronger in Sweden than in many other countries [17]. Cyber victimization has been shown to have negative outcomes similar to those of TBV, for example, psychosomatic complaints [19, 20], depressive symptoms [4, 10, 20–23], anxiety [20], loneliness [24], lower self-rated health (cyber victimization included in written–verbal bullying victimization) [12], lower self-esteem [4, 6, 20], lower academic performance [20], substance use [21], delinquency [21], self-injury [10], suicidal ideation [10, 20, 23], and suicide attempts [10]. The highest psychological distress has been seen among children who are victimized in both contexts [10].

Social support is a protective factor for health [25], associated with a lower prevalence of both cyber victimization [2, 26] and TBV [2, 27–30]. Parents are the first significant source of support for children, and parental support continues to be valuable [29, 31], even though peer support becomes increasingly important as children grow older [18, 28]. A meta-analysis of studies on parenting behavior and peer victimization concluded that positive parenting behavior including good communication of parents with the child, a warm and affectionate relationship, parental involvement and support, and parental supervision were protective against peer victimization [30]. Results from a longitudinal study showed that family support protected adolescents living in single-parent families from cyber victimization when their friends were not supportive, and furthermore that low family support coupled with low friend support predicted the highest levels of cyber victimization [26].

Social support is furthermore associated with a lower prevalence of mental health problems in adolescents [27–29, 31–33]. Communication with parents is fundamental in establishing the family as a protective factor [18], and young people who easily communicate with their parents have fewer SHC [33]. Although relationships to parents have been shown to be a stronger predictor of good health than relationships to siblings or friends in adolescence [33, 34], positive peer relationships are crucial for adolescents regarding developmental tasks such as forming identity, developing social skills, and establishing autonomy [18].

The way social support influences health can be described by two alternative (but not mutually exclusive) theoretical models: the main effect model and the stress-buffering model [25]. According to the main effect model, support has an overall beneficial effect on psychological outcomes, regardless of the level of adversity experienced. In the context of the present study, social support would reduce SHC among students irrespective of exposure to cyber harassment. According to the stress-buffering (or interaction) model, the protective effect of social support differs according to the level of stress experienced. In this context, the beneficial effect of social support on SHC would vary among students differently exposed to cyber harassment (CH) (statistically there would be a significant interaction effect of social support and CH on SHC) [25, 35].

Earlier research on TBV among children has investigated these two models for different sources of social support on a variety of mental health outcomes. Solid evidence for the main effect model has been provided [27–29, 32, 36–38], but evidence regarding the stress-buffering model is inconclusive. While several studies have reported support for stress-buffering effects on different combinations of social support and gender [28, 29, 32, 37], others have found no support for the stress-buffering model [36, 38]. The effect of social support on cyber victimization and mental health outcomes has been less extensively researched. To the best of our knowledge there is no earlier study on adolescent cyber victimization that has explored the theories of main and stress-buffering effects of support from parents and friends with respect to SHC. We found one population-based study (in which cyber victimization was included in written-verbal bullying) that reported that the opportunity to speak to an adult about things that worried the child modified the associations between cyber victimization and self-reported general health [12]. The present study will primarily contribute to the existing body of knowledge by adding information on the effect of support from parents/friends on the association between cyber victimization (measured as harassment) and SHC. In this study cyber victimization is defined as “cyber harassment” instead of “cyberbullying” in order to include even single incidents of cyber violation during the past year.

We hypothesize that there will be significant associations between CH and SHC among 9th grade students in Scania, with stronger associations for having been cyber harassed several times than for only once (H1). We also hypothesize that there will be a generally beneficial effect of parental/friend support (a main effect) on the association between CH and SHC (H2). Furthermore, we hypothesize that there will be indications of a stress-buffering effect of social support on the association between CH and SHC (H3), however, we make no assumptions regarding differences between parental/friend support or gender differences, due to inconsistent findings in earlier research. Finally, we hypothesize that further adjustment for TBV in the multiple adjusted regression models will weaken the association between CH and SHC slightly, but will not affect the significance of the association. This result would indicate that CH has an effect of its own on SHC (H4).

## Methods

### Study population and procedure

A large public health survey of children and adolescents was performed in Skåne (Scania), the southernmost region of Sweden, in 2012. The main purpose of the survey was to map out the health situation among adolescents, and the questionnaire included questions on living conditions, lifestyle factors, mental and physical health, sleep, well-being, social relations, and school [39]. The students were informed of the purpose of the survey, that participation was voluntary, that their answers would remain confidential, and that the results of the survey would be used in research. Their parents were likewise informed and invited to inform the teachers if they did not want their children to participate. The questionnaires were completed anonymously during one school-hour in classrooms during one week in March 2012. Students with reading disabilities had access to technical help to complete the questionnaire. Nearly 30000 students answered the questionnaires in grades 6 and 9 and the second year of upper secondary school (i.e., adolescents around 12, 15, and 17 years of age), including 9792 students in 9th grade (response rate 83 %). The selected study sample for the present research study consists of 9th grade students with answers on all eight SHC items; that is 8544 students, 4190 boys (49.0 %) and 4354 girls (51.0 %). This study was reviewed and approved by the Regional Ethical Committee at Lund University, Sweden (Dnr 2013/317). Written parental consent was not required, as 9th grade students are viewed as mature enough to make their own decision regarding participation in this type of public health survey in Sweden.

### Measurements

#### Dependent variable: subjective health complaints

*Subjective health complaints* is a general term used to describe a variety of common health symptoms such as headache, stomachache, nervousness, and so on, experienced with or without a diagnosis [40]. We chose to assess SHC by the Health and Behaviour in School-aged Children Symptom Checklist (HBSC-SCL), a reliable and valid instrument [40] used for decades in the cross-national WHO collaborative study Health Behaviour in School-aged Children [18]. The students were asked how often they had experienced the following eight health complaints in the last six months: headache, stomachache, backache, feeling low, feeling irritable or bad tempered, feeling nervous, difficulties in getting to sleep, and dizziness [41, 42]. Each health complaint was rated on a five-point frequency scale, ranging from one point for “Rarely or never” to five points for “About every day,” generating an index score of 8–40, with higher scores indicating more SHC [42]. Cronbach’s alpha coefficient in the present study was 0.81 for both boys and girls, respectively. SHC for boys were mean 15.8, median 15, mode 12, and for girls mean 19.8, median 19, mode 16.

#### Independent variables

*Cyber harassment* was assessed by the question “Have you during the past 12 months, in school or out of school, been exposed to harassment or violation involving a cell phone and/or the Internet (text messaging, instant messaging (MSN), Facebook, e-mail or similar)?” The response options were “No”, “Yes, once” and “Yes, several times” [39, 43].

*Social support * was measured with a question on parental/friend support which was phrased “If you have a problem or just want to talk to someone, how easy or difficult would it be to talk to…?” Several alternative sources of social support were given, including “Parents or the adults you live with” and “Friends.” There were five response options for each alternative, ranging from “Very easy” to “Very difficult.” The response options were dichotomized into “Easy communication” (“Very easy”, “Rather easy”), and “Not easy communication” (“Neither easy nor difficult,” “Rather difficult,” “Difficult”). “Easy communication” equals high support and “Not easy communication” equals low support. This question has been used for many years in a large national survey of Swedish 9th grade students on alcohol, tobacco, and drug use [44].

#### Covariates

Adjustment was made for the following potential confounders: *Parental occupation* (both/one/no parent working) [12, 31]; *Parental origin* (both/one/no parent born in Sweden) [12]; *Daily smoking* (smoking cigarettes every day/less often) [45]; *Intense alcohol consumption* (drinking a large quantity in one session at least once a month/drinking alcohol less often) [13, 44, 46]; and *Disability* (no disability versus any disability of the following alternatives: hearing disability/visual disability that cannot be corrected by glasses or contact lenses/moving disability/reading–writing disability, dyslexia/ADHD-ADD/other disability.) [12]. Further adjustment was made for *Traditional bullying victimization* in an additional analysis, assessed by the question” How often have you been bullied in school during the past few months?” Those who had been bullied two or three times a month or more often (i.e., more than once a month) during the past few months were categorized as traditional bullying victims in line with earlier research [6, 18, 41]. *Body weight* (BMI normal weight: boys <23.29; girls <23.94; overweight: boys 23.29–28.29; girls 23.94–29.10, BMI obesity: boys 28.30+, girls 29.11+ [12, 47]. All analyses were stratified according to gender, as there are known gender differences regarding SHC (girls report more SHC) [18, 31, 33, 41] as well as social support (in Sweden more 15-year old boys than girls report easy communication with parents [18], while adolescent girls have been known to report more peer support [28, 29]).

### Statistics

Differences in background characteristics were analyzed by Pearson chi square tests for all categorical variables, and by one-way ANOVA for SHC.

To examine the associations between cyber harassment and SHC modified by support, a series of hierarchical regression analyses were performed according to the procedures recommended by Baron and Kenny [35]. In Model 1, the dependent variable of SHC was regressed on the independent variable of CH, adjusted for age, parental occupation, parental origin, daily smoking, intense alcohol consumption, and disability (H1). BMI was not included in the multiple adjusted analyses as there were no significant associations between body weight and CH in our study sample. In Model 2, parental/friend support was added (with separate analyses for the two types of support) (H2). In a final third model, the interaction of CH and social support was added (separate analyses for the two types of support). If the interaction term added in Model 3 was statistically significant, a moderating (or stress-buffering) effect of social support on the association between CH and SHC could be inferred (H3). Furthermore, an identical series of hierarchical regression analyses was performed with additional adjustment for TBV (H4). The statistical analyses were performed using IBM SPSS Statistics version 22.

## Results

Descriptive statistics of the study population stratified by exposure to cyber harassment is presented in Table 1. Among boys, 540 (14 %) had experienced CH during the past year: 351 boys (9 %) once and 189 boys (5 %) several times. The prevalence was higher among girls; 849 girls (20 %) reported that they had been cyber harassed during the past year: 562 girls (13 %) once and 287 girls (7 %) several times. Victimization by CH was significantly more often reported by boys and girls who did not have two working parents, who smoked and had intense alcohol consumption, had some form of disability, and who did not find it easy to talk to parents or friends if having a problem (low parental/friend support).

**Table 1:** Characteristics (%) of cyber harassed 9th grade boys and girls. The Scania public health survey among children and adolescents, 2012

	Boys			*p*-value^a^	Girls			*p*-value^a^
	Cyber harassed past year				Cyber harassed past year			
	No (*n* = 3372; 86 %)	Yes, once (*n* = 351; 9 %)	Yes, several times (*n* = 189; 5 %)		No (*n* = 3333; 80 %)	Yes, once (*n* = 562; 13 %)	Yes, several times (*n* = 287; 7 %)	
Parental occupation								
Both parents working	83.5	78.8	75.3		80.4	74.8	74.6	
One parent working	13.7	17.2	18.4		15.7	21.4	18.7	
No parent working	2.8	4.1	6.3	0.007**	3.9	3.9	6.7	0.003**
Parental origin								
Both parents born in Sweden	67.2	70.1	63.8		65.6	69.2	67.8	
One parent born in Sweden, one abroad	11.9	9.9	16.2		11.3	13.2	15.5	
Both parents born abroad	20.9	20.1	20.0	0.288	23.2	17.5	16.6	0.002**
Daily smoking								
No	95.1	91.1	77.3		94.8	90.3	83.8	
Yes	4.9	8.9	22.7	0.000***	5.2	9.7	16.2	0.000***
Intense alcohol consumption								
No	85.7	77.3	68.9		87.5	79.0	73.0	
Yes	14.3	22.7	31.1	0.000***	12.5	21.0	27.0	0.000***
Weight								
Normal weight	77.1	73.3	71.2		88.6	89.3	85.9	
Overweight	19.1	22.4	24.7		9.5	9.5	10.3	
Obese	3.8	4.3	4.1	0.257	1.8	1.2	3.8	0.136
Disability								
No	77.4	67.3	58.5		80.1	72.0	65.0	
Yes	22.6	32.7	41.5	0.000***	19.9	28.0	35.0	0.000***
Bullied traditionally more than once a month								
No	97.5	91.6	71.5		97.8	94.5	76.8	
Yes	2.5	8.4	28.5	0.000***	2.2	5.5	23.2	0.000***
Easy to talk to friends if problems								
Yes	77.2	72.4	66.0		80.8	77.8	74.6	
No	22.8	27.6	34.0	0.000***	19.2	22.2	25.4	0.017*
Easy to talk to parents if problems								
Yes	68.5	55.2	47.9		63.1	52.0	46.2	
No	31.5	44.8	52.1	0.000***	36.9	48.0	53.8	0.000***
SHC-index 8–40^b^								
Mean	15.2	17.6	20.8	0.000***	18.9	22.1	24.8	0.000***
Median	14	17	20		18	22	25	
SD	5.3	5.5	7.8		6.0	6.1	6.8	

Significance levels: *p < 0.05, **p < 0.01, ***p < 0.001

^a^Pearson chi-square test for all variables except SHC-index

^b^One-way ANOVA

CH was significantly more often reported by boys and girls who had experienced traditional bullying victimization (TBV) during the past few months. The overlap between past year CH and past few months TBV increased with increasing exposure to CH; among those who had been cyber harassed several times, 29 % of boys and 23 % of girls reported TBV, compared to around 2 % of boys and girls who had not been cyber harassed. The numbers should be interpreted with care, as both definitions and time frames of the two types of victimization differ, but a pattern of increasing simultaneous victimization can still be discerned. The total prevalence of TBV during the past few months was 4 % among boys and girls, respectively (data not shown).

The results of multiple hierarchical linear regressions assessing main and interaction (stress-buffering) effects of social support on the relationship between CH and SCH are presented in Table 2 (boys) and Table 3 (girls). Having been cyber harassed once or several times during the past year was associated with higher levels of SHC, controlling for age, parental occupation, parental origin, daily smoking, intense alcohol consumption, and disability (Model 1 in Tables 2 and 3). The associations were stronger for CH several times than for CH once, supporting H1. Including parental/friend support in the next model revealed a negative association between support and SHC, indicating a main effect of social support on SHC in boys and girls, supporting H2. Furthermore, the levels of SHC were somewhat decreased, but remained statistically significant (Model 2 in Tables 2 and 3). Adding interaction variables in the final stage of the analysis revealed different patterns for boys and girls (Model 3 in Tables 2 and 3). Among boys there was a significant interaction effect between parental support and CH several times, indicating a stress-buffering effect of parental support on SHC for boys who had been cyber harassed several times (Model 3 in Table 2). Friend support showed significant interactions with both categories of CH for boys, with stronger influence on SHC for CH several times than CH once. Among girls there were no significant interactions between either type of support and CH (Model 3 in Table 3). Thus, H3 was partially supported; interaction effects were found for boys but not for girls. In an additional analysis with further adjustment for TBV, the association between CH and SHC was only slightly affected and remained statistically significant (Additional file 1: Table S1 (boys) and Additional file 2: Table S2 (girls)), supporting H4.

**Table 2:** Estimated regression coefficients (95 % confidence intervals (CI)) for the association between cyber harassment (CH), parental/friend support, and subjective health complaints (SHC) among 9th grade boys in Sweden

	Model 1	Model 2		Model 3	
		Parental support	Friend support	Parental support	Friend support
Predictors	Regression coefficients (95 % CI)				
CH past year					
No					
Yes, once	2.2*** (1.5–2.8)	1.9*** (1.2–2.5)	2.1*** (1.4–2.7)	1.2* (0.2–2.1) *p* = 0.013	3.3*** (2.1–4.5)
Yes, several times	4.6*** (3.8–5.5)	4.2*** (3.4–5.1)	4.5*** (3.7–5.3)	5.2*** (4.0–6.4)	6.2*** (4.7–7.7)
Social support		−2.5*** (−2.9 to–2.1)	−1.6*** (−2.0 to −1.2)	−2.5*** (−2.9 to −2.1)	−1.3*** (−1.7 to −0.8)
Interaction					
CH once x support				1.2 (−0.07 to 2.4) *p* = 0.064	−1.6* (−3.0 to −0.2) *p* = 0.022
CH several times x support				−2.0* (−3.6 to −0.3) *p* = 0.018	−2.5** (−4.2 to −0.7) *p* = 0.007
Adjusted R Square	0.087	0.133	0.101	0.135	0.104

Model 1 excludes social support, Model 2 includes social support, and Model 3 includes cyber harassment-social support interactions. All models controlled for age, parental occupation, parental origin, daily smoking, intense alcohol consumption, and disability

*p < 0.05; **p < 0.01; ***p < 0.001

**Table 3:** Estimated regression coefficients (95 % confidence intervals (CI)) for the association between cyber harassment (CH), parental/friend support, and subjective health complaints (SHC) among 9th grade girls in Sweden

	Model 1	Model 2		Model 3	
		Parental support	Friend support	Parental support	Friend support
Predictors	Regression coefficients (95 % CI)				
CH past year					
No					
Yes, once	2.4*** (1.8–3.0)	2.1*** (1.6–2.7)	2.4*** (1.8–2.9)	2.1*** (1.3–2.9)	2.4*** (1.1–3.6)
Yes, several times	4.8*** (4.1–5.6)	4.4*** (3.7–5.2)	4.7*** (3.9–5.5)	4.3*** (3.3–5.4)	4.4*** (2.8–6.0)
Social support		−2.5*** (−2.9 to −2.1)	−2.6*** (−3.0 to −2.1)	−2.5*** (−3.0 to −2.1)	−2.6*** (−3.1 to −2.0)
Interaction					
CH once x support				0.02 (−1.1 to 1.1) *p* = 0.974	−0.1 (−1.4 to 1.4) *p* = 0.988
CH several times x support				0.2 (−1.3 to 1.7) *p* = 0.805	0.4 (−1.4 to 2.2) *p* = 0.681
Adjusted R square	0.13	0.167	0.156	0.167	0.155

Model 1 excludes social support, Model 2 includes social support, and Model 3 includes cyber harassment-social support interactions. All models controlled for age, parental occupation, parental origin, daily smoking, intense alcohol consumption, and disability

*p < 0.05; **p < 0.01; ***p < 0.001

**Fig. 1:**
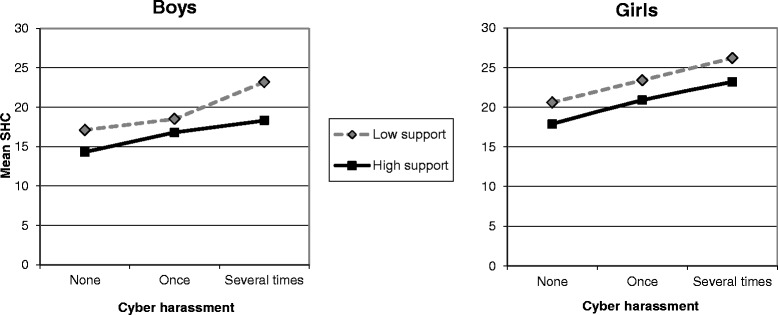
Mean level of subjective health complaints (SHC) by cyber harassment stratified by parental support. Past year cyber harassment (none/once/several times) in 9th grade boys and girls with high/low parental support (measured as communication). The Scania public health survey among children and adolescents, 2012

**Fig. 2:**
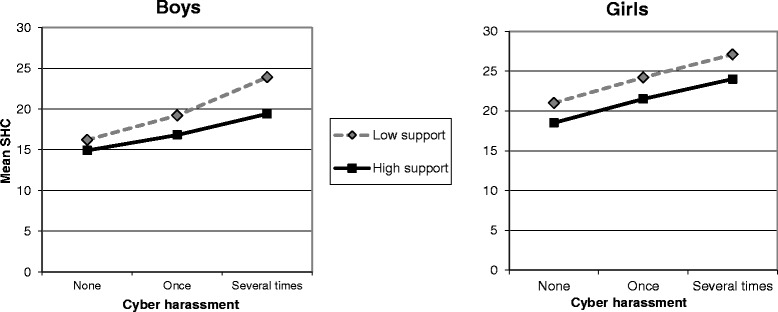
Mean level of subjective health complaints (SHC) by cyber harassment stratified by friend support. Past year cyber harassment (none/once/several times) in 9th grade boys and girls with high/low friend support (measured as communication). The Scania public health survey among children and adolescents, 2012

The mean level of SHC by CH stratified by social support is illustrated in Fig. 1 (Parental support) and Fig. 2 (Friend support). The mean level of SHC increased with increasing exposure to CH among both boys and girls. A generally beneficial (main) effect of support on the association between CH and SHC is visualized by a higher line representing low support compared to a lower line representing high support among boys and girls. Among boys, the increases in SHC were steeper between CH once and several times for boys with low parental support (Fig. 1), and gradually steeper for those with low friend support (Fig. 2), in comparison with the respective lines representing high support, indicating an interaction (stress-buffering) effect of both types of support on the association between CH and SHC among boys. Among girls, the almost parallel lines representing high and low support illustrate the absence of an interaction effect (Figs. 1 and 2).

## Discussion

The present study showed that having been cyber harassed during the past year was associated with higher levels of SHC in adolescent boys and girls, with stronger associations for cyber harassment (CH) several times than CH once (H1). Girls were more often cyber harassed than boys, which is in line with most studies [2, 6, 9, 10, 13–15, 19, 22, 24, 48], but not all [4, 11, 23, 26]. Perhaps CH can be seen as an extension of relational bullying which is more common among girls? [8]. In agreement with earlier research, girls also reported higher levels of SHC [18, 31, 33, 41] as well as more peer support [28, 29].

The protective influence of parental and friend support (measured as communication) against SHC in the context of peer victimization (CH) was investigated according to the main effect model and the stress-buffering model [25, 35]. Similar research has been conducted earlier on traditionally bullied children, but as far as we know, this is the first study on cyber victimized adolescents exploring main and stress-buffering effects of support from parents or friends on SHC.

Evidence was found for a generally beneficial effect (main effect) of both parental and friend support on the association between CH and SHC in both genders (H2). Furthermore, indications of a stress-buffering effect were seen for both parental and friend support among cyber harassed boys, while there were no indications of a stress-buffering effect for either type of support among girls (H3). These findings are in line with an earlier study on traditional bullying victimization (TBV), which found main effects for social support (parents, teachers, classmates, close friend) on depression among both boys and girls, and furthermore, a stress-buffering effect of parental and close friend support among peer-victimized boys [29]. The generally beneficial (main) effect of social support on psychosocial outcomes among victimized children has been consistently shown in earlier research on TBV [27–29, 32, 36–38], but findings regarding stress-buffering effects differ. Some earlier studies have reported stress-buffering effects among girls [32], and boys [29], respectively, some studies have reported stress-buffering effects among both genders [28, 37], while yet other studies have found no evidence of a stress-buffering effect [36, 38]. In the present study, stress-buffering effects of parental and friend support were seen among boys, but not among girls. It has been suggested that gender differences in stress-buffering effects of social support could be due to mediating factors, such as different coping styles among boys and girls [29]. Earlier studies have shown that girls are more likely than boys to seek social support when faced with online problematic situations [49]. Seeking social support could be defined as both an emotion-focused and a problem-focused coping strategy, depending on the content of the social support received [50]. Social support is a broad concept covering several different aspects, such as communicating that a person is valued and accepted by others, thereby enhancing self-esteem (esteem support), helping the person to understand and cope with stressors (informational support), providing distraction from worries and social belonging (social companionship), and providing time and material support (instrumental support) [25]. The present study measured support as communication, which in a good relationship could be a proxy for all the above-mentioned aspects of support. However, in the present study we do not know the content of the support received. Girls have been shown to use more emotion-focused and ruminative coping than boys [51], and emotion-focused coping has been shown to be associated with more health complaints and depressive feelings among cyberbullied children [52]. Girls also report using more problem-focused coping than boys, but it is possible that these attempts at problem solving are less effective because rumination interferes [53]. There is evidence that boys recover faster than girls from the negative effects of victimization on symptoms of anxiety, depression, and self-esteem after cessation of victimization [54]. Perhaps boys benefit more from the support they do get and are more often encouraged to use distraction to cope with peer victimization [29].

One study found a significant mediating effect instead of a moderation effect of social support on depressive feelings among traditionally bullied children, with different patterns among boys and girls [55]. Victimized boys received very little support and hence suffered depression, while the mediation effects were more diffuse among girls and did not pertain so much to the type of involvement in bullying as to the subsequent lack of support. The present study did not investigate mediation effects, but it was much more common among cyber harassed boys to lack support of a close friend: 20 % of boys and 6 % of girls who had been cyber harassed several times had no close friend, compared to 6 % of boys and 4 % of girls not cyber harassed (data not shown). However, additional adjustment for close friend in analyses on friend support did not significantly affect the associations between CH and SHC or the interaction patterns among boys and girls (data not shown).

It is noteworthy that cyber victims do not always seek help from others, and when they do, they prefer friends over adults [1, 7, 8]. Usually, only a minority of parents are told [7, 8], so the protective effect of easy communication with parents is probably due more to a generally supportive and caring relationship (main effect) than to specific communication about the cyber incident. Children prefer to discuss online problems with friends, as they fear that parents will invade their privacy or limit their online freedom [3, 49]. Having more friends has been shown to be protective in traditional bullying, but not in cyberbullying [2]. It may still be that adolescents find greater support in peers than in parents when negative experiences involve peers [28].

In line with earlier research [1, 4, 6, 8, 10, 11, 19, 20, 22] there was a substantial overlap of CH and traditional bullying victimization. It has been debated whether the negative effects of cyber victimization in reality might be due to the negative effects of simultaneous TBV [6, 11]. In the present study, further adjustment for TBV did not change the associations with SHC substantially, which indicates that victimization by CH has an effect of its own on SHC (H4). These results are in line with other cross-sectional studies [22, 23, 48] as well as a longitudinal study [50] showing evidence for a unique contribution of cyber victimization to psychological distress over and above the contribution of TBV. However, a large longitudinal Finnish study found that electronic victimization only leads to increases in depression when combined with TBV [11]. In this study the prevalence of electronic-only victimization was as low as 0.5 % (and the prevalence of combined electronic and traditional bullying victimization was 1.4 %), by a strict definition of cyber victims as being targeted more than once a month during the past couple of months. The researchers concluded that electronic-only victims seemed to be selected on a different basis than those targeted traditionally, that is, from among the relatively well-adjusted and socially accepted students who might have better coping skills to start with. The victim groups are thus defined differently in this study compared with the present study (which used a much wider definition) and probably differ in composition.

### Strengths and limitations

A strength of the present study is the large total population sample including the majority of the 9th graders in the county of Scania, which generates good statistical power and reduces selection bias. Another strength is the use of an outcome measure (HBSC-SCL) that has been widely used and is well validated [18, 40]. Furthermore, the data set included information on several potential confounding factors, such as parental occupation and origin, risk behavior (smoking and alcohol drinking), disability, and traditional bullying victimization (TBV). However, there were also some limitations to the present study. First, due to the cross-sectional nature of the survey, we cannot make causal inferences on the true associations between cyber harassment (CH) and SHC. Second, only one general question on cyber victimization was used, asking for “cyber harassment” and not for “cyberbullying”, with different time frames for CH and TBV (past 12 months and past few months, respectively). Unresolved issues regarding how to define and measure cyber victimization complicate cross-study comparisons as well as comparisons between cyber victimization and TBV. The question on CH is new and has not been extensively validated [43]. Although harassment may be a broader concept than bullying, having been cyber harassed only once during the past year still showed significant associations with SHC. Even a short duration of being a cyber victim may have severe effects, given the potentially wide audience and the permanence of messages [1, 8]. In the present study there was no question on perpetration of peer victimization, which means that we do not know how many cyber victims were also harassing others in cyberspace, and bully-victims are known to have the poorest health outcomes compared to bullies, victims, and non-involved [1, 19, 20, 27]. The intensity and duration of bullying are important for the consequences of victimization [52, 55], but we had information only on frequency (once/several times) of CH and not on duration. Furthermore, we had no information on risky online behavior (such as posting personal information, and photos, and using a webcam to chat with strangers), which has shown significant associations with cyber victimization [4, 13].

The present study was a step in the direction of clarifying the moderating role of social support in cyber harassed adolescents. However, future research should delve deeper into what aspects of social support really matter, with further investigations regarding the observed gender differences. It is important and urgent to reach consensus on a definition of cyber victimization in future research. Agreeing on a static and comprehensive definition is, however, a challenging task, rendered even more difficult by the rapid advances in communications technology [10].

## Conclusions

In conclusion, victimization by cyber harassment is prevalent and associated with higher levels of SHC in 9th grade adolescents in Scania. Support from parents and friends (measured as easy communication) has a generally beneficial (main) effect for both boys and girls, while indications of a stress-buffering effect of parental and friend support were seen among boys only. Intervention programs focusing on the mechanisms behind peer victimization, aiming at improving the quality of peer and family relationships among children and adolescents, might reduce the incidence of victimization (from both traditional bullying and cyber harassment) and lower the prevalence of psychosomatic complaints among the young [11, 24, 32, 34, 41].

## Additional files

Additional file 1: Table S1. Estimated regression coefficients (95 % confidence intervals (CI)) for the association between cyber harassment, parental/friend support, and subjective health complaints (SHC) among 9th grade boys in Sweden, additionally adjusted for traditional bullying victimization. (DOC 17 kb) Additional file 2: Table S2. Estimated regression coefficients (95 % confidence intervals (CI)) for the association between cyber harassment, parental/friend support, and subjective health complaints (SHC) among 9th grade girls in Sweden, additionally adjusted for traditional bullying victimization. (DOC 17 kb)

## Abbreviations

SHC: Subjective Health Complaints
HBSC-SCL: Health and Behaviour in School-aged Children Symptom CheckList
CH: Cyber Harassment
TBV: Traditional Bullying Victimization
