# Supplementary material for: Subjective health complaints in adolescent victims of cyber harassment: moderation through support from parents/friends - a Swedish population-based study
Source: BMC Public Health. 2015 Sep 23;15:949. doi: 10.1186/s12889-015-2239-7 (PMC4581473; doi:10.1186/s12889-015-2239-7)
Supplement: Additional file 2: Table S2. — Estimated regression coefficients (95 % confidence intervals (CI)) for the association between cyber harassment, parental/friend support, and subjective health complaints (SHC) among 9th grade girls in Sweden, additionally adjusted for traditional bullying victimization. (DOC 17 kb) [file 12889_2015_2239_MOESM2_ESM.docx]

Additional Table 2. Estimated regression coefficients (95 % confidence intervals (CI)) for the association between cyber harassment, parental/friend support, and SHC among 9th grade girls in Sweden, additionally adjusted for traditional bullying victimization.
